# Impaired cerebrospinal fluid transport due to idiopathic subdural hematoma in pig: an unusual case

**DOI:** 10.1186/s12917-021-02954-2

**Published:** 2021-07-20

**Authors:** Nagesh C. Shanbhag, Nicholas Burdon Bèchet, Marios Kritsilis, Iben Lundgaard

**Affiliations:** 1 Department of Experimental Medical Science, Lund University, Sölvegatan 17, BMC A1304, 223 62 Lund, SE Sweden; 2 Wallenberg Centre for Molecular Medicine, Lund University, Lund, Sweden

**Keywords:** Cerebrospinal fluid transport, Cisterna magna infusion, Glymphatic system, Pig, Subdural hematoma

## Abstract

**Background:**

We report the effects of the presentation of an idiopathic subdural hematoma (SDH) in an adult domestic pig on the glymphatic system, a brain-wide solute clearance system. This accidental finding is based on our recently published study that described this system for the first time in large mammals. Our current results define the need to investigate cerebrovascular pathologies that could compromise glymphatic function in gyrencephalic animal models as a tool to bridge rodent and human glymphatic studies.

**Case presentation:**

The pig underwent intracisternal infusion of a fluorescent tracer under general anesthesia to delineate cerebrospinal fluid (CSF) pathways, and was euthanized at the end of 3 h of tracer circulation. During brain isolation, a hematoma measuring approximately 15 × 35 mm in size beneath the dura was evident overlying fronto-parietal brain surface. Interestingly, CSF tracer distribution was markedly reduced on dorsal, lateral and ventral surfaces of the brain when compared with a control pig that was infused with the same tracer. Furthermore, regional distribution of tracer along the interhemispheric fissure, lateral fissure and hippocampus was 4–5-fold reduced in comparison with a control pig. Microscopically, glial-fibrillary acidic protein and aquaporin-4 water channel immunoreactivities were altered in the SDH pig brain.

**Conclusions:**

This is the first case of impaired glymphatic pathway due to an idiopathic SDH in a pig. Potential etiology could involve an acceleration-deceleration injury inflicted prior to arrival at our housing facility (e.g., during animal transportation) leading to disruption of bridging veins along the superior sagittal sinus and impairing CSF pathways in the whole brain. This accidental finding of globally impaired glymphatic function sheds light on a novel consequence of SDH, which may play a role in the enhanced cognitive decline seen in elderly presenting with chronic SDH.

## Background

The glymphatic system is a newly discovered brain-wide solute clearance system that serves brain waste removal via perivascular-mediated cerebrospinal fluid (CSF) flux, draining along perivenous spaces and ultimately exiting the brain into nasal and cervical lymphatic systems [1–3]. This system was first described in 2012 and is most active during sleep, whereat CSF moves from the subarachnoid space (SAS) down perivascular spaces (PVS) surrounding arteries, finally penetrating and cleaning the brain parenchyma of waste, including amyloid β [1, 4, 5]. The movement of CSF from the PVS through the brain is facilitated by and dependent on aquporin-4 (AQP4) water channels which are polarized at the astrocytic endfeet that project to form the outer boundary of the PVS [1, 4]. Additionally, cerebral arterial pulsations and brain vasomotion contribute to driving glymphatic-based solute clearance across the CSF spaces [6–8] .

To study glymphatic transport, fluorescently labelled tracers have been widely utilized in animal models and are infused into the CSF compartment (e.g., cisterna magna, CM) to delineate the perivascular CSF pathways [9–11]. Impaired glymphatic transport has been shown to occur in animal models of traumatic brain injury, ischemic stroke, and subarachnoid haemorrhage [12–15]. However, most glymphatic studies have been carried out in rodents and thus to achieve translational relevance we have recently set up and described the glymphatic pathways in a porcine model using state-of-the-art imaging techniques [11, 16]. The porcine glymphatic pathways appear very similar to those described in rodents, most importantly the penetration of CSF tracer into the brain occurs via PVS bounded externally by AQP4 water channels [11]. A key difference in pigs is that the gyrencephalic nature of the brain facilitates robust CSF transport via the sulci, and interestingly the density of PVS in the pig brain is 4-fold greater than in mice, highlighting the more extensive glymphatic architecture presumed necessary for waste removal in a larger brain [11].

Herein, we describe a follow-up case of an adult male Landrace pig which presented with an idiopathic subdural hematoma (SDH). So far, no such similar reports have been described in the literature. This pig underwent a refined investigation to study the glymphatic system by injecting a CSF tracer in the CM. When compared with a healthy control pig we uncovered marked global reductions in glymphatic function which may be of great importance in adding to our understanding of the cognitive decline seen in SDH patients.

## Case presentation

A 52-kg, male Landrace pig (*Sus scrofa domesticus*) purchased from an approved supplier was housed at the animal facility of the Biomedical Centre, Lund University for 3 days prior to a glymphatic experiment at an ambient temperature of 22–25 °C and 30–70% humidity on a 12 h light-dark cycle. The pig had ad libitum access to drinking water. The animal had been maintained in accordance with the ethical guidelines approved by the Malmö-Lund ethical Committee on Animal Research (Jordbruksverket Dnr 5.2.18–05527/19) and complied with the ARRIVE guidelines for reporting of animal experiments.

The intention of the study was to investigate glymphatic pathways in a porcine model. The pig referred to as the control pig (55-kg male Landrace) was along with the SDH pig part of a larger study. Both pigs underwent the same tracer injections leading to the inclusion of the control pig as a representative for normal porcine glymphatic function. Animals underwent overnight fasting with access to only drinking water. On the day of surgery, animals were anesthetized as described previously [11]. Briefly, animals were premedicated with Zoletil and Dexdomitor intramuscularly with general anesthesia maintained using a triple drip of ketamine, fentanyl and midazolam [11]. Physiological parameters [heart rate (HR), blood pressure (BP), body temperature (BT), pCO_2_] were continuously recorded during the entire procedure. The intervention consisted of an infusion of fluorescently labelled tracer (bovine serum albumin (BSA)-Alexa647, 65 kDa, A34785, Invitrogen) via CM. For this, a total infusion volume of 500 μl of the tracer was administered at the rate of 100 μl/min and allowed to circulate for 3 h prior to euthanization with intravenous pentobarbital (140 mg/kg). Small volumes of tracer based on brain size when introduced into the CSF have been shown not to produce sustained elevations in intracranial pressure (ICP) [17, 18].

**Fig. 1:**
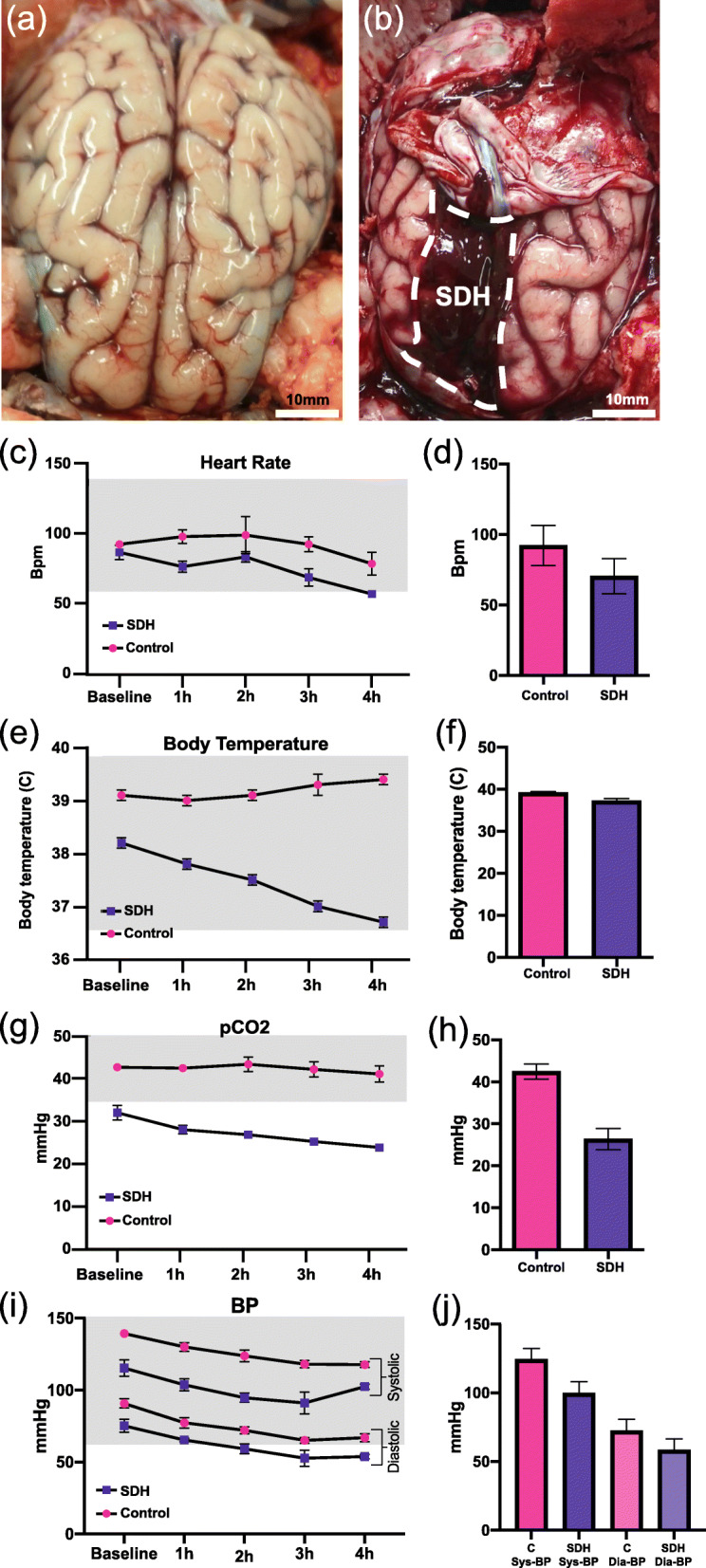
Hematoma location and perioperative physiological parameters. (**A**) Control versus (**B**) idiopathic subdural hematoma (SDH) pig brains. Time course and average values of (**C-D**) heart rate, (**E-F**) body temperature, (**G-H**) partial pressure of CO_2_, and (**I-J**) blood pressure as assessed during the whole intervention. C, control; Sys BP, systolic blood pressure; Dia-BP, diastolic blood pressure. Shaded regions in panel (**C**), (**G**), (**E**) and (**I**) represent normal range of respective parameters [12, 13]. Data represented as mean ± standard deviation (SD)

Upon necropsy of the pig, after euthanization, the dorsal skull cap was carefully removed to isolate the whole brain intact. Upon gross examination after the reflection of the dorsal dura, an idiopathic subdural hematoma was found, characterized by visual inspection and palpation as lying beneath the dura but above arachnoid and pia mater (SDH, dark red in color). The SDH measured 15 × 35 mm overlying the fronto-parietal brain surface beneath the superior sagittal sinus (Fig. 1A, B). The appearance and color of hematoma beneath the dura mater was indicative of a long-standing bleed which may have compromised the intracranial homeostasis. Although HR, BT, and BP from both the pigs were within normal physiological parameters, pCO2 was found to be lower than normal range (HR: 60–140 bpm; BT: > 36.6 °C; BP: > 90/> 60 mmHg; pCO2: 35–55 mmHg) in SDH pig [15, 19] (Fig. 1C-J). Subsequently, the whole brain was extracted and fixed in 4% paraformaldehyde for processing. Directly upon brain removal from the cranial vault it became apparent through inspection that minimal amounts of tracer had entered the cranial space and was instead seen emerging from the subarachnoid space surrounding the spinal cord.

**Fig. 2:**
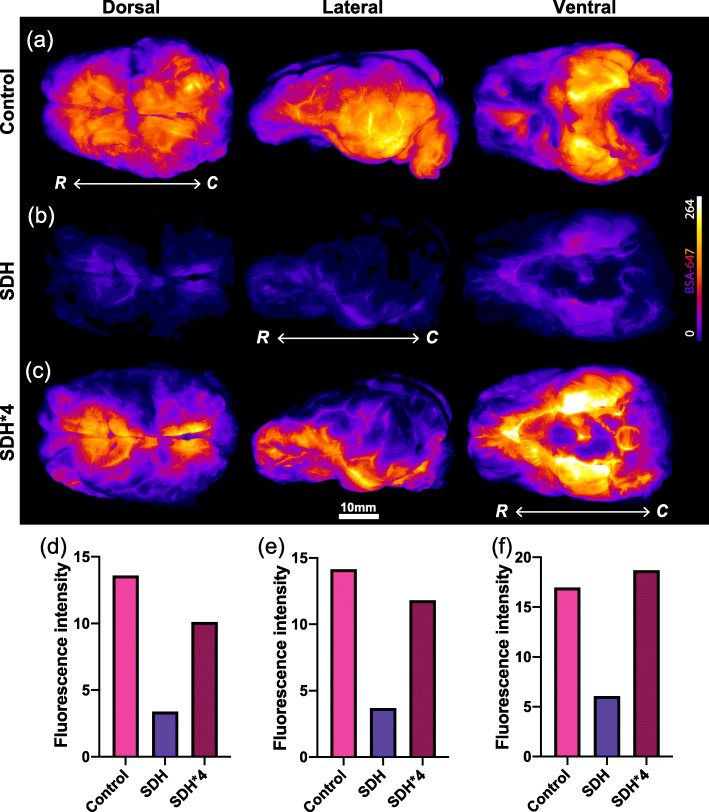
Macroscopic CSF tracer distribution on the whole brain surface. Fluorescent tracer (BSA-Alexa647) distribution along the dorsal, lateral and ventral surface of the brain between control (**A**) and SDH pig (**B**), and (**C**) upon enhancing the signal exposure value by a factor of 4 (SDH*4) in relation to the control pig. Surface tracer intensities along (**D**) dorsal, (**E**) lateral, and (**F**) ventral surface of the brain between control, SDH and SDH*4 (normalized) conditions. Intensity values are represented as arbitrary units. CSF, cerebrospinal fluid; BSA-Alexa647, bovine serum albumin-AlexaFluor647; SDH, subdural hematoma. R, rostral; C, caudal

To make inferences on glymphatic function, in this case glymphatic influx into the brain, the mean fluorescent intensity generated by the tracer within the brain tissue was quantified and analyzed. The movement of the tracer into the brain acts as representative measure of CSF movement into the brain thus permitting the assessment of glymphatic influx. We first examined the tracer signal intensity at dorsal, lateral, and ventral surfaces of the brain using a stereo microscope (Nikon SMZ25) with a Plan Apo 0.5x objective (0.08 NA) equipped with an Andor Zyla 4.2 Plus sCMOS camera (Mag-0.75x, Zoom-1.5x). Macroscopic findings in the SDH pig showed a marked reduction in the overall tracer intensities over dorsal, lateral, and ventral brain surfaces when compared with the control pig that was injected with the same tracer (Fig. 2A-C). Interestingly, when the imaging exposure value was increased by a factor of 4 (SDH*4), the tracer signal intensity appeared to yield comparable levels with the control pig (Fig. 2A-F). When examining the SDH*4 data, the CSF pathways on the dorsal surface appeared to be most compromised as compared to lateral and ventral surfaces (Fig. 2D-F).

**Fig. 3:**
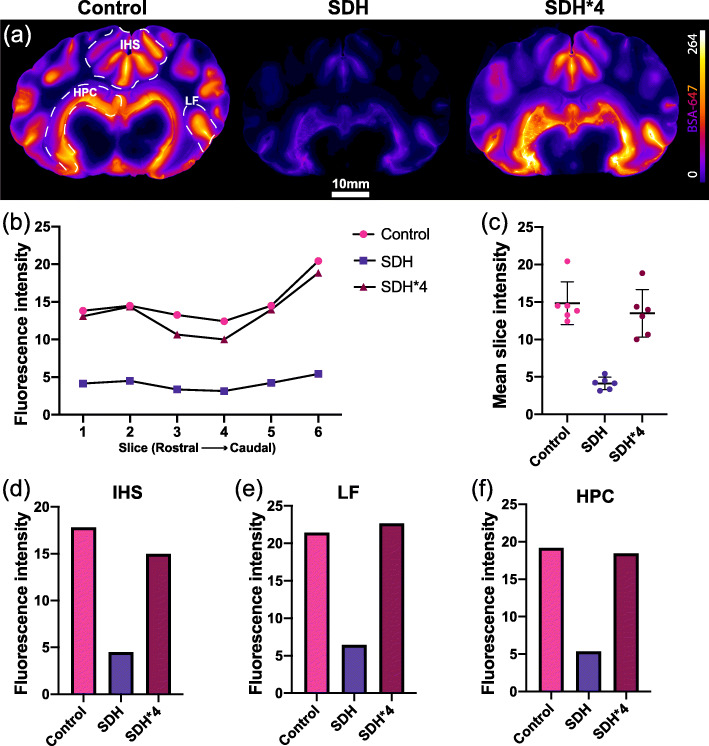
Regional CSF tracer distribution in the whole brain slices. (**A**) Representative images of whole brain slices depicting tracer (BSA-Alexa647) distribution in control, SDH and SDH*4 (normalized) conditions. (**B**) Tracer intensity plots from 6 whole brain slices along the rostral-caudal axis. (**C**) Mean intensities of all the 6 slices combined. Region specific mean tracer intensities along the (**D**) interhemispheric sulci (HIS), (**E**) lateral fissure (LF), and (**F**) hippocampus (HPC) between control, SDH and the normalized conditions. CSF, cerebrospinal fluid; BSA-Alexa647, bovine serum albumin-AlexaFluor647; SDH, subdural hematoma. Data represented as mean ± standard deviation (SD)

To capture a two-dimensional view of the impact of SDH on glymphatic tracer influx, we coronally sectioned the whole brain into 10 mm thick slices which were also imaged with a stereo microscope. As with the whole brains, the SDH coronal sections exhibited a noticeable reduction in glymphatic tracer influx which appeared to normalize if the imaging exposure time was once again quadrupled (SDH*4) from the standard control exposure time (Fig. 3A). A rostral-caudal plot of slice tracer intensities showed a drastic reduction in intensity across all slices in the SDH brain (Fig. 3B). Interestingly, the pattern of tracer distribution along the rostral-caudal axis was still maintained despite the SDH, which can be better appreciated in the SDH*4 data (Fig. 3B). Analysis of all slices batched showed a 3–4 fold reduction in tracer intensity which was normalized in the SDH*4 group (Fig. 3C). Specific regions of interest including the interhemispheric sulcus (IHS) and lateral fissure (LF), which we have shown previously to be important structures for initial CSF distribution, and the hippocampus (HPC), which gives insight into subcortical glymphatic influx, were further investigated and exhibited 4–5 fold reductions in tracer signal intensity in the SDH pig again with near-normalization in the SDH*4 data (Fig. 3D-F). These findings suggest that the space occupying lesion (in our case, SDH) impaired the previously reported CSF distribution sites (e.g., IHS, LF) and subsequent glymphatic tracer influx.

**Fig. 4:**
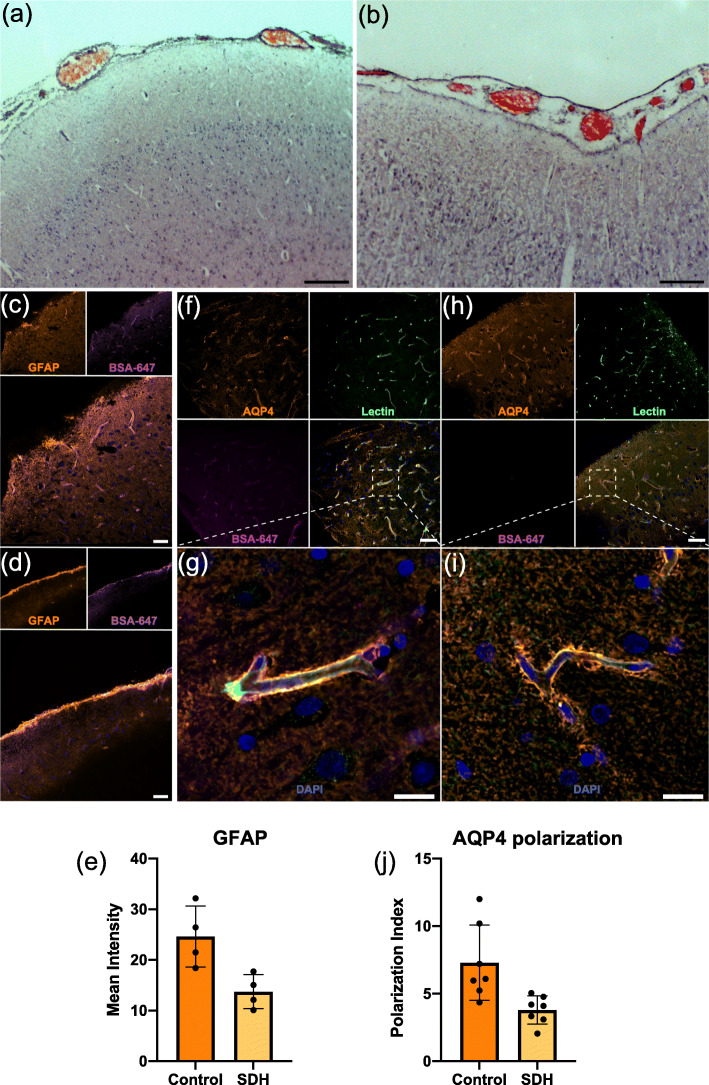
Microscopic investigation shows astrocyte dysfunction. (**A-B**) Representative images of hematoxylin and eosin staining of brain cortical surface and pial vessels from a control and an SDH pig, respectively. Scale bars = 2 mm. (**C-D**) Representative images of GFAP immunostaining (orange, astrocytes) of a control and an SDH pig, respectively, with BSA-647 tracer (magenta). Scale bar = 50 μm. (**E**) Quantification of the mean GFAP intensity in control and SDH pigs, *n* = 4 cortical regions analyzed in *N* = 1 animal per group. (**F-I**) Representative images of AQP4 immunostaining (orange, astrocyte water channel) and lectin (green, blood vessels) staining, with BSA-647 tracer (magenta) of a control (**F-G**) and an SDH pig (**H-I**). Scale bars (**F, H**) = 50 μm. Scale bars (**G, I**) = 10 μm. (**J**) Quantification of AQP4 polarization in a control and an SDH pig, *n* = 7 vessels analyzed per animal in *N* = 1 animal per group. AQP4, aquaporin-4; BSA-Alexa647, bovine serum albumin-AlexaFluor647; GFAP, glial-fibrillary acidic protein; SDH, subdural hematoma. Data represented as mean ± standard deviation (SD)

For microscopic investigation, parts of the dorsal cortex underlying the SDH and corresponding regions from the control pig were cut into 100 μm sections using a vibratome (Leica VT1200S). Hematoxylin and eosin staining revealed no observable anomalies in the surface pial vessels beneath the SDH (Fig. 4A-B). Since glymphatic function has been shown to be highly dependent on astrocytes, we further carried out immunohistochemical staining for glial-fibrillary acidic protein (GFAP) and AQP4 to investigate astrocyte integrity (Fig. 4C-J). Overall GFAP intensity was found to be reduced in the SDH animal indicating some level of astrocyte dysfunction (Fig. 4C-E). Furthermore, AQP4 polarization (relative AQP4 intensities at vascular surface versus whole tissue) which is important for normal glymphatic function also showed a trend for being lower in the SDH pig (Fig. 4F-J). Thus, in addition to the pressure-mediated consequences of SDH on glymphatic function it appears as if underlying astrocytic insult may too have contributed to the observed glymphatic impairment.

## Discussion and conclusions

To the best of our knowledge, this is the first reported case of impaired glymphatic function in the context of a subdural hematoma in a large mammal. Etiology of the subdural hematoma is unclear in our case, however, a common etiology for a SDH presentation is from an acceleration-deceleration injury i.e. “whiplash” resulting in disruption of bridging veins along the superior sagittal sinus [20]. Thus, it is plausible that during transport from the supplier to the lab that the pig may have suffered such an injury had the transport vehicle rapidly changed speed without the animal’s head being tied down or braced in some form. This is further supported by the absence of any contusions on the skin or skull both overlying the SDH and throughout the cranium in general pointing to an absence of blunt force trauma. In contrast, an acceleration-deceleration injury may account for the absence of any overt contusions in the context of a SDH presentation [20].

A SDH represents a space occupying lesion within the cranial vault and based on size it will result in increased intracranial pressure (ICP) [21, 22]. In order to compensate for the SDH and resultant ICP perturbation, it stands to reason volumetric compensation in the form of reduced rostral CSF movement would amount and downstream of this reduced glymphatic influx [23]. Furthermore raised ICP has been shown to cause astrocyte dysfunction which could further impair glymphatic function [24]. From our findings, we speculate that the hematoma led to a sustained increase in the ICP and thus led to a pressure-mediated re-routing of CSF, and subsequently tracer, down the spinal cord and away from the brain leading to compromised cerebral glymphatic function. Secondary to this, it appears as if the suspected raised ICP led to astrocyte perturbations and a reduced AQP4 polarization which is known to be important for normal glymphatic function [1, 25]. Additionally, the hematoma could have altered the cerebrovascular pulsatility and vasomotion, both of which play an important role in the perivascular CSF solute clearance [6–8].

These alterations together are sufficient to have led to the observed impairment in the CSF tracer transport and glymphatic influx. This coincidental finding could be of great importance in intimating the consequences of sustained periods of reduced glymphatic function and how this could impact overall brain health in cases of undiagnosed chronic SDH, which are widely reported in the elderly [26–28]. One of the main etiologies for SDH has been head trauma which is a risk factor for long-term neurodegenerative conditions [29, 30]. Thus, our finding may explain how an impaired glymphatic function because of SDH, downstream of head trauma, may contribute to the neurodegeneration as observed in Alzheimer’s disease [31].

In summary, we present a unique case of idiopathic subdural hematoma in a 52-kg pig, which resulted in an impaired global distribution of CSF tracer in the brain. In order to avoid using compromised animals for future experiments, it will be of value to implement neuroimaging (e.g., non-contrast head CT scan) prior to CNS experiments, including glymphatic interventions [32]. Finally, these findings beg the question of what unknown glymphatic-based consequences arise in the brains of patients impacted by silent SDH and if this puts these patients at risk for neurodegenerative diseases.

## Data Availability

Not applicable.

## Abbreviations

AQP4: Aquaporin-4
BSA-Alexa647: bovine serum albumin-Alexa647
BP: blood pressure
BT: body temperature
CM: cisterna magna
CSF: cerebrospinal fluid
CT: computed tomography
GFAP: Glial-fibrillary acidic protein
HPC: hippocampus
HR: heart rate
IHS: interhemispheric sulcus
LF: lateral fissure
pCO_2_: partial pressure of carbon dioxide
SDH: subdural hematoma
